# Superconducting Self-Shielded and Zero-Boil-Off Magnetoencephalogram Systems: A Dry Phantom Evaluation

**DOI:** 10.3390/s24186044

**Published:** 2024-09-18

**Authors:** Keita Tanaka, Akihiko Tsukahara, Hiroki Miyanaga, Shoji Tsunematsu, Takanori Kato, Yuji Matsubara, Hiromu Sakai

**Affiliations:** 1 Department of Science and Engineering, Tokyo Denki University, Saitama 350-0394, Japan; tsukahara@mail.dendai.ac.jp; 2 Sumitomo Heavy Industries, Ltd., Yokosuka 237-0061, Japan; hiroki.miyanaga@shi-g.com (H.M.); shoji.tsunematsu@shi-g.com (S.T.); takanori.kato@shi-g.com (T.K.); yuji.matsubara@shi-g.com (Y.M.); 3 Faculty of Science and Engineering, Waseda University, Tokyo 169-8555, Japan; hsakai@waseda.jp

**Keywords:** biomagnetism, magnetoencephalogram, magnetic shielding, zero-boil-off system

## Abstract

Magnetoencephalography (MEG) systems are advanced neuroimaging tools used to measure the magnetic fields produced by neuronal activity in the human brain. However, they require significant amounts of liquid helium to keep the superconducting quantum interference device (SQUID) sensors in a stable superconducting state. Additionally, MEG systems must be installed in a magnetically shielded room to minimize interference from external magnetic fields. We have developed an advanced MEG system that incorporates a superconducting magnetic shield and a zero-boil-off system. This system overcomes the typical limitations of traditional MEG systems, such as the frequent need for liquid helium refills and the spatial constraints imposed by magnetically shielded rooms. To validate the system, we conducted an evaluation using signal source estimation. This involved a phantom with 50 current sources of known location and magnitude under active zero-boil-off conditions. Our evaluations focused on the precision of the magnetic field distribution and the quantification of estimation errors. We achieved a consistent magnetic field distribution that matched the source current, maintaining an estimation error margin within 3.5 mm, regardless of the frequency of the signal source current. These findings affirm the practicality and efficacy of the system.

## 1. Introduction

To understand high brain functions such as cognition, memory, and attention, it is necessary to measure neural activity in the cerebral cortex. Among various neuroimaging techniques, magnetoencephalography (MEG) stands out due to its high temporal resolution in the millisecond range and adequate spatial resolution in the centimeter range. This makes MEG particularly effective for tracking activity in specific brain regions, making it ideal for functional brain mapping during cognitive tasks [1]. In contrast, other widely used methods like functional magnetic resonance imaging (fMRI) and electroencephalography (EEG) face limitations in either temporal or spatial resolution, respectively [2,3]. Consequently, MEG is a superior tool for elucidating the precise temporal and spatial dynamics of neural activity that underlie cognitive processes. Neuronal activity in the cerebral cortex involves the flow of intracellular currents through dendrites when neurons are activated. According to the corkscrew rule, a MEG system measures the weak magnetic field generated by these currents. An intracellular current of 0.2 pAm implies that MEG can measure 5.0 nAm of intracellular current, which is equivalent to 2.5 × 10^4^ synchronously active neurons (current dipole) [4]. This ability to quantify the activity of a large number of neurons enables researchers and clinicians to gain insights into the dynamics of neural networks and understand how different brain regions communicate and coordinate during various cognitive processes and behaviors.

A typical MEG system requires a large amount of liquid helium to maintain the superconducting quantum interference device (SQUID) sensor in a stable superconducting state. However, liquid helium is expensive and difficult to obtain. To address this challenge, recent advancements have introduced MEG systems that utilize optically pumped magnetometers (OPMs). These OPM sensors eliminate the need for liquid helium cooling, making them lightweight and portable. As a result, measurements can be taken while subjects are in a natural state of motion. Furthermore, OPM-MEG systems are easy to set up and offer high spatial resolution. However, they also present technical challenges such as sensitivity to ambient noise and sensor heat generation [5]. Furthermore, because the magnetic field generated by the brain is extremely weak (10^−12^–10^−15^ T), a magnetically shielded room is required for both conventional MEG and OPM-MEG to reduce interference from external fields. A magnetically shielded room is generally a cube or cuboidal of approximately 27 m^3^ (approximately 3.0 m on each side) with from two to four layers of permalloy alloy, a material with high magnetic permeability, and one layer of copper or aluminum to shield the room from environmental magnetic fields. Additionally, there are limitations on its installation location; for example, there should be no elevators or cars nearby that generate loud magnetic noise. Conventional 3 m scale magnetically shielded rooms typically weigh more than 10,000 kg, requiring integration into the building’s foundation. This substantial weight creates a significant constraint, especially for installations above ground floors. In contrast, the MEG system we have developed, which incorporates a superconducting magnetic shield, is much lighter than conventional shielded rooms. This reduction in weight alleviates installation constraints, allowing the system to be more easily installed in locations other than ground floors. This advantage is particularly beneficial for installations in limited spaces or existing buildings. Furthermore, OPM-MEG systems often require shields that reduces background magnetic fields as much as possible. However, our developed system uses a superconducting magnetic shield, which eliminates the need for a zero-field environment. This simplifies the overall system design and provides a significant advantage in terms of installation flexibility.

In these MEG system designs, we developed a full-head 64-channel MEG system equipped with a superconducting magnetic shield and a zero-boil-off system [6]. In zero-boil-off operating conditions for helium, the system recondenses the generated gas back into liquid, minimizing losses as the liquid helium vaporizes. This MEG system does not require a magnetically shielded room or liquid helium filling. Furthermore, by using a superconducting magnetic shield rather than a magnetically shielded room, the system is anticipated to offer improved shielding against environmental noise in the low-frequency band. In this study, we present the results of evaluating the signal-source accuracy of this system by varying both the intensity and frequency of the signal-source current using a dry phantom under zero-boil-off operating conditions.

## 2. MEG Systems

**Figure 1:**
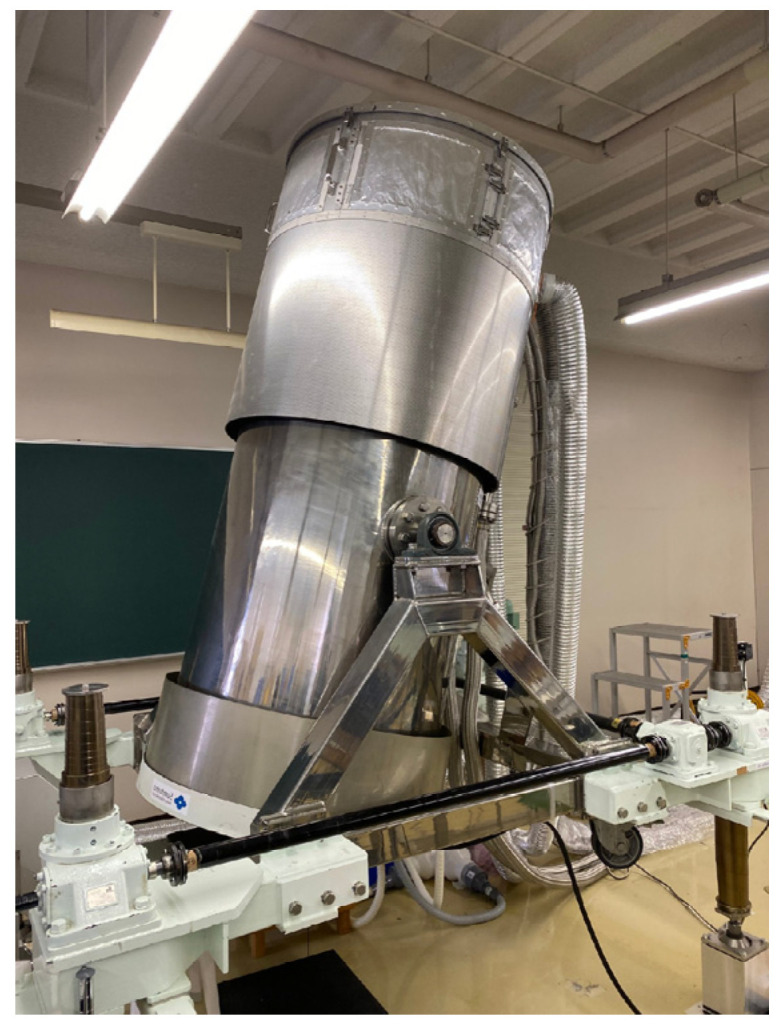
Superconducting self-shielded and zero-boil-off MEG systems.

**Figure 2:**
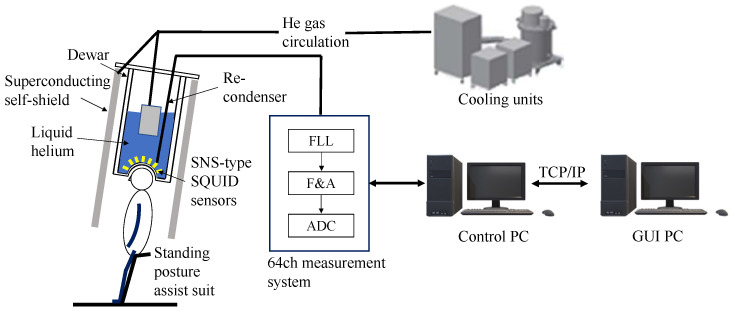
Schematic of the zero-boil-off system for cooling the superconducting magnetic shield and recondensing the evaporated He gas in a 64-channel MEG measurement system. FLL denotes a flux-locked loop circuit, F&A refers to filter and amplifier circuits, and ADC stands for analog-to-digital converter. This setup does not use a magnetically shielded room.

Figure 1 and Figure 2 show an actual photograph and the configuration of the MEG system, respectively. A high-temperature superconducting magnetic shield (HTSMS) uses the perfect antimagnetic property of superconductors (Meissner effect) by maintaining their superconducting state with gas cooled with a refrigerator. In this work, the overall configuration comprised a MEG measurement system, an HTSMS, and a zero-boil-off cooling system. The MEG system was 2.6 m long, 1.9 m wide, 2.3–2.8 m high, and weighed 2000 kg.

**Figure 3:**
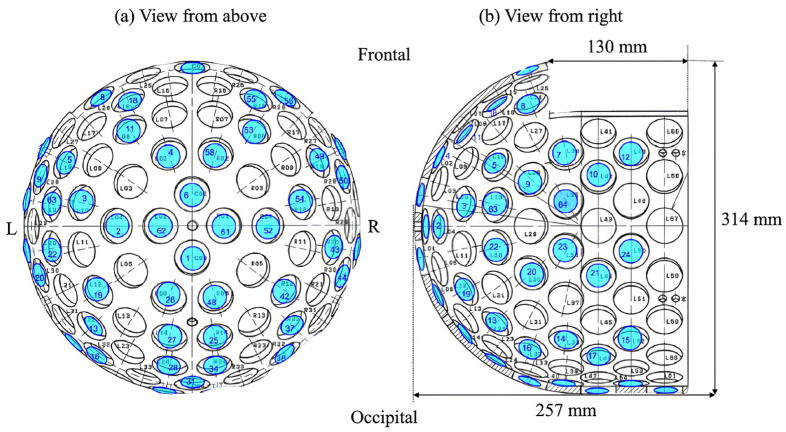
SQUID sensor arrangement of the MEG system (64 sensors). (**a**) Shows the view from the top of the head, and (**b**) shows the right temporal view. Blue circles indicate the locations of the sensors. The total number of spaces available for sensor installation is 128.

The MEG sensor employed a superconductor/normal-metal/superconductor (SNS) junction-type SQUID sensor (first-order axial gradiometer). Unlike general SQUID sensors, which use a superconductor/insulator/superconductor (SIS) tunnel Josephson junction, the SNS junction avoids the low-frequency noise, also known as telegraphic noise, commonly found in devices with insulating layers in SIS tunnel junctions [7]. This makes it particularly suitable for MEG measurements in the low-frequency range (below 10 Hz). The detector coil had a diameter of 30 mm and a baseline of 45 mm. Currently, the system features 64 sensors, with the potential to be expanded to 128 channels. Figure 3 depicts the sensor arrangement, with the layout shown from the parietal and right temporal views. The left side mirrors the placement of the right side.

The MEG system was installed in a typical classroom in a university facility. A bus depot and an elevator were located approximately 10 m from the classroom, a highway approximately 1 km away, and a railroad line approximately 3 km away. Table 1 shows a comparison between the specification of the developed HTSMS-MEG and general SQUID type MEG.

### 2.1. Superconducting Magnetic Shield

A magnetic shield can serve as an alternative to a magnetically shielded room. In this setup, a high-temperature superconductor, (Bi, Pb) 2Sr_2_Ca_2_Cu_3_O_x_ (Bi2223), was used to create a cylindrical shield around the subject. This shield was fabricated using the thermal spraying method, with a diameter of 650 mm and a height of 1600 mm. The measured critical temperature of Bi2223 was 103 K. The superconducting surface of the shield was engineered to sustain a critical current density of at least 100 A/cm^2^, providing a shielding factor of 5000 at the center of the sensor array, even at a low frequency of 1 Hz. The HTSMS performed well at lower frequencies compared to traditional magnetically shielded rooms, particularly at frequencies below 10 Hz [7]. The superconducting magnetic shields were expected to efficiently mitigate environmental magnetic noise from sources such as automobiles and trains, even when they peaked at frequencies below 1 Hz.

### 2.2. Zero-Boil-Off Cooling System

**Figure 4:**
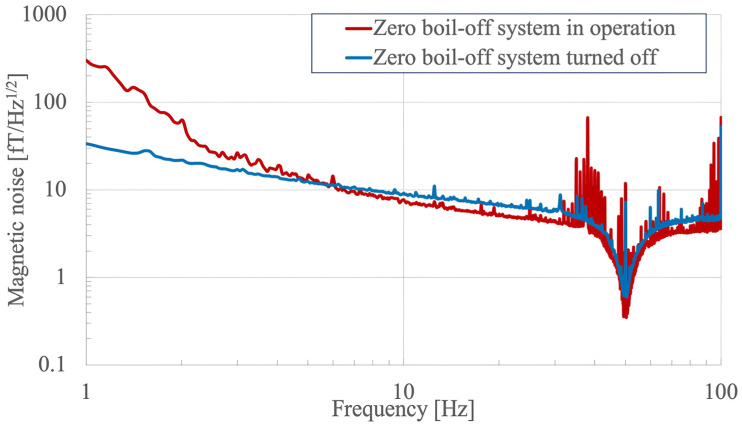
Spectrum density of the environmental magnetic noise measured using the superconducting magnetically shielded MEG system with (red line) and without (blue line) the zero-boil-off system. Note that the spectral density is the average of all 64 sensors. A notch filter with a utility frequency of 50 Hz was used during the measurement.

The cooling system with the zero-boil-off feature employed a two-module setup. It utilized a Joule–Thomson mechanism in conjunction with a dual-stage Gifford–MacMahon (GM) system to cool the SQUID sensors to below 4.5 K. A recondenser was employed to convert the evaporated helium gas back into liquid form. Additionally, an auxiliary cooling system was designed with a circulation loop including a heat exchanger and a dual-stage GM system, responsible for maintaining the temperature of the radiation shield of the Dewar and the HTSMS below 90 K. The GM coolers, which had moving parts that could generate noise, were located remotely. The innovative zero-boil-off technology eliminated the daily 12 L helium evaporation from the Dewar and operated with minimal acoustic disturbance during measurements. Typically, in MEG systems with this technology, noise at a specific frequency around 1 Hz and its harmonics, originating from the compressor of the cooling system, as well as low-frequency noise from the helium liquefaction process, are present. Nevertheless, this system maintained a low-noise output even when the zero-boil-off feature was active. Figure 4 shows the spectral density of the environmental magnetic noise measured by the superconducting magnetically shielded MEG system with and without the zero-boil-off system. Note that the spectral density is the average of all 64 sensors. A notch filter with a utility frequency of 50 Hz was used in the measurement conditions. Several peaks were observed from 35 to 50 Hz when the zero-boil-off system was in operation, likely related to the cooling system.

### 2.3. Evaluation by Dry Phantom

**Figure 5:**
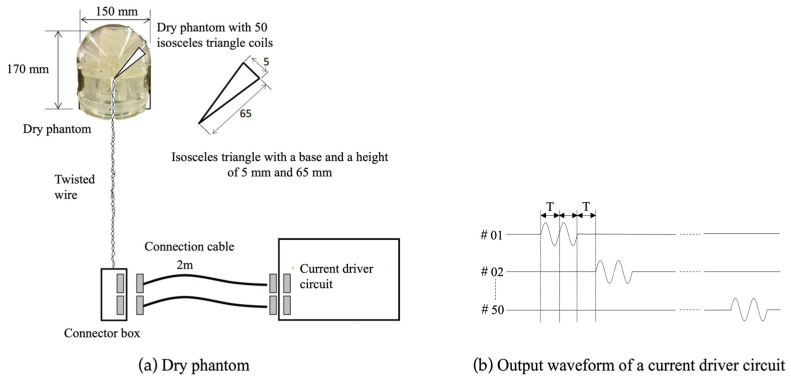
Block diagram of a dry phantom generating 50 current dipoles (**a**) and output waveforms from the current driver circuit (**b**), where T in this figure (**b**) represents the period corresponding to the frequency of the sinusoidal wave. “#” denotes the number of the stimulation coil.

To assess the accuracy of the signal source estimation, an experimental study was conducted using a phantom with a known true position and temporal activity in the dipole. This approach is valuable for quantitatively evaluating and validating MEG systems, as it enables investigations into the impact of errors in head positioning and noise in the data on the accuracy of the estimation [8,9]. To evaluate the accuracy of the MEG with a phantom, the MEG signal generated using an artificial current dipole was measured using our MEG system, and the signal source was estimated. The position, direction, and intensity of the artificial current dipole were assumed to be known. The evaluation was based on the discrepancies in the signal source estimation results. A dry-type phantom from the Kanazawa Institute of Technology was used to generate artificial current dipoles [10,11]. This phantom comprised 50 isosceles triangular coils (height 65 mm, base 5 mm), each producing an equivalent current dipole. Figure 5 shows the block diagram of the dry phantom and the output waveforms from the current driver circuit. The dry phantom was 150 mm in diameter and 170 mm high, and was placed so that it was tangent to the sensor at the top of the head in Figure 3 and the center of the head of the phantom. The output sine wave from the current driver circuit can oscillate two wavelengths with a period corresponding to the specified frequency. The amplitude can be set to 50 nAm and 100 nAm for the current dipole.

### 2.4. Signal Source Estimation Method

The current dipoles were 50 and 100 nAm, and the frequencies of the sinusoidal signals were 3 Hz (delta), 6 Hz (theta), 11 Hz (alpha), 20 Hz (beta), and 40 Hz (gamma). The amplitude of a transient response (response with a latency of 100 ms, N1m) with a typical auditory cortex as the source was approximately 50 nAm [12]. The MEG value for each current dipole intensity was measured, added, and averaged 70 times. The signal source was estimated using the minimum-norm current estimation method using MNE-Python [13] for the waveform after bandpass filtering (2–45 Hz) to minimize the effect of environmental magnetic noise.

## 3. Results

### 3.1. Magnetic Field Distribution

**Figure 6:**
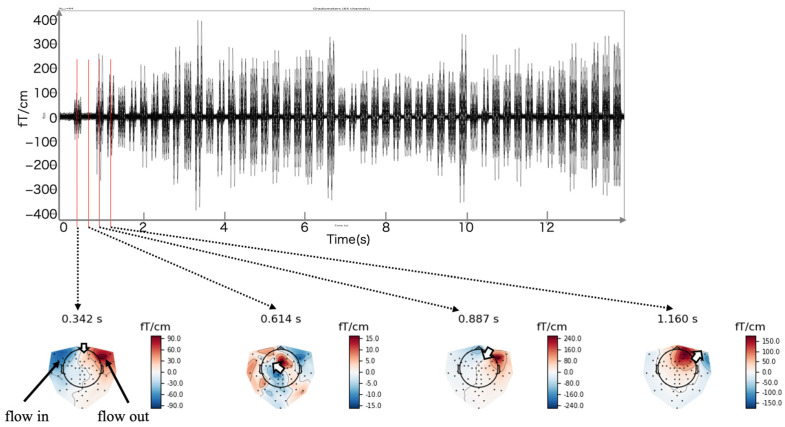
(**Top**) An average waveform and (**bottom**) the magnetic fields corresponding to the current dipole measured at a current dipole of 100 nAm applied to each coil. The red area indicates the flux out, whereas the blue area indicates the flux in. Arrows indicate the direction of the estimated current dipole.

Figure 6 illustrates the MEG waveforms measured with a current dipole of 100 nAm applied to the coil. The lower part of the figure shows the magnetic field distribution corresponding to the current dipole, with red indicating the outward flux and blue indicating the inward flux. The direction of the current dipole can be determined using the corkscrew rule applied to the magnetic field distribution. The magnetic field distribution calculated using the measured MEG values was consistent with the expected distribution for all 50 current dipoles and current dipoles (50 and 100 nAm) generated with the coils in the phantom.

### 3.2. Source Estimation Error

**Figure 7:**
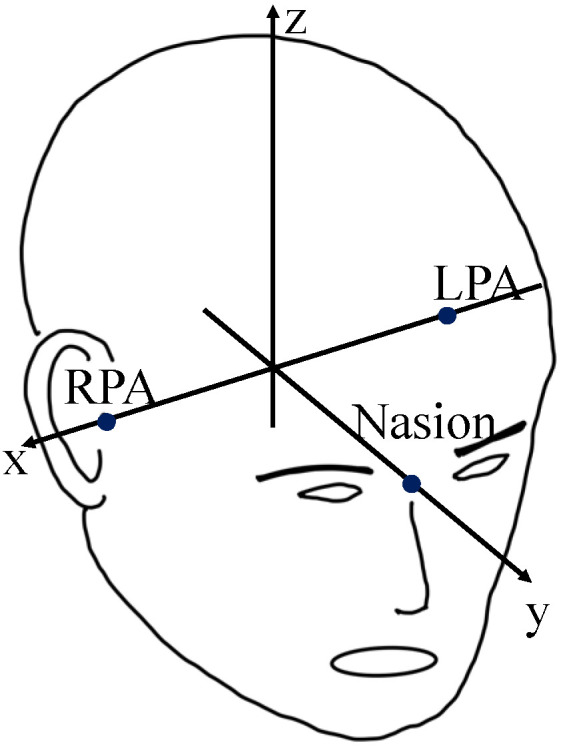
Definition of the three axes (x, y, and z coordinates) for the signal source estimation.

**Figure 8:**
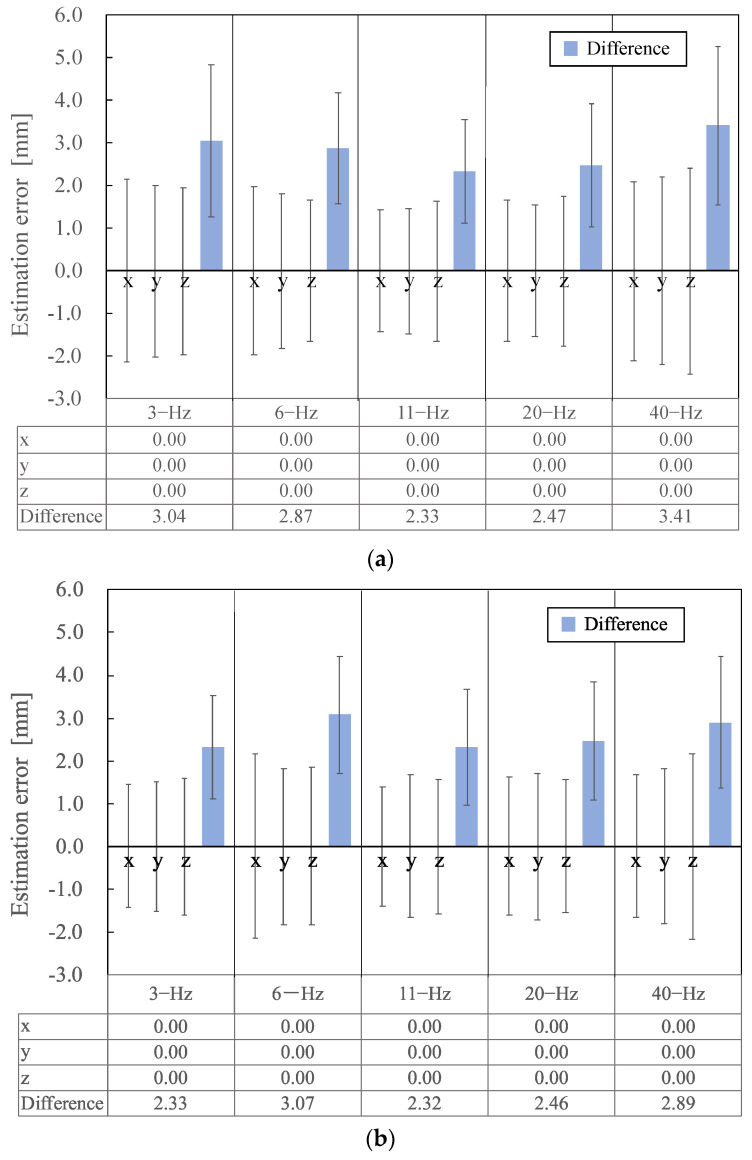
Signal source estimation results showing the mean differences between the positions of the 50 current dipoles for current dipoles of (**a**) 50 nAm and (**b**) 100 nAm along the three axes (x, y, and z coordinates) and the absolute values of the current dipoles on the three axes (blue bars).

Figure 7 schematically defines the three axes along which the signal source estimation was performed. Figure 8 shows the mean differences between the estimated and actual positions of the 50 current dipoles (50 nAm and 100 nAm) on each axis and the absolute values of the current dipoles on the three axes (blue bars) as estimation errors. The figure presents absolute mean values, with estimation errors between 3.5 mm for a current intensity of 10 μA and 3.1 mm for 100 nAm.

Table 2 presents the mean estimation error (mm) for 50 current sources categorized into five brain regions, illustrating the differences in source estimation errors across these regions. The table includes results for current source frequencies of 3, 6, 11, 20, and 40 Hz, and current dipole of 50 and 100 nAm. The findings indicate that the estimation error was relatively large in the frontal and occipital regions (mean: frontal 3.45 mm, occipital 3.23 mm) compared to the left-temporal (2.62 mm), right-temporal (2.30 mm), and parietal (2.10 mm) regions. There was no significant difference in estimation error between the current dipoles of 50 nAm (mean: 2.84 mm) and 100 nAm (mean: 2.63 mm).

### 3.3. Goodness of Fit by Number of Additive Averages

**Figure 9:**
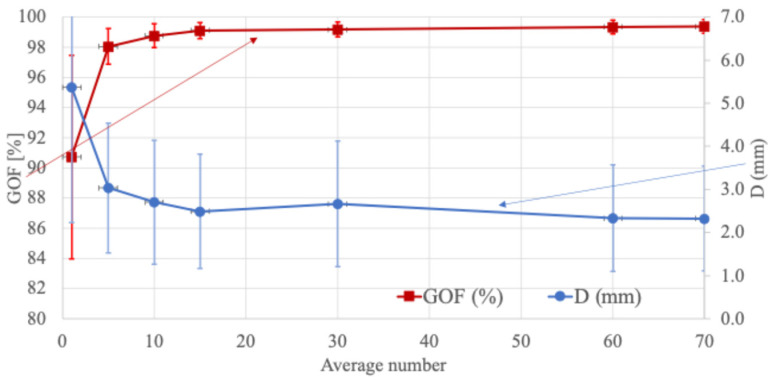
Estimation error and goodness of fit (GOF) results for the number of additions at a current dipole of 50 nAm. D indicates the estimation error.

Figure 9 presents the evaluation results based on the average number of additions. The red points represent the goodness of fit (GOF), indicating an agreement between the estimated current dipole and the measured magnetic fields, and the estimation error as a function of the number of additions for a current dipole of 50 nAm. A GOF value of 90% or higher is generally considered acceptable. The results show that the GOF value reached 96%, with an estimation error of less than 2.5 mm after averaging 10 values. The findings indicate that for the transient response of the auditory cortex (N1m), 80–100 additive averages are sufficient for accurate estimation.

## 4. Discussion

To evaluate the performance of the MEG with the zero-boil-off system, we used a phantom to create an artificial current dipole. This setup allowed us to estimate the signal source from MEG waveforms and examine estimation errors. Initially, we verified the magnetic field distribution produced by the artificial current dipole. Figure 6 shows that the magnetic field distribution, including flux out and flux in, accurately corresponds to the direction of the current dipole, demonstrating a precise magnetic field distribution even with the zero-boil-off system active.

The dipole estimation error results shown in Figure 8 indicate that the average values for the three axes (x, y, and z coordinates) of the signal source, estimated from the 50 artificial current dipoles, were zero for both 50 and 100 nAm. This suggests no physical misalignment (bias) of the phantom, indicating the proper alignment of the MEG sensor coordinate system with the head coordinate system (the phantom coordinate system).

The estimation error in this study represents the difference between the true position of the current dipole and the average of the measured values, reflecting high measurement accuracy. The typical spatial resolution, defined as the shortest distance over which two simultaneously active brain areas can be distinguished, is reported to be between around 3.0–5.0 mm [14] and 2.0–3.0 mm [15] for MEG systems, allowing for the precise monitoring of cortical activity. Spatial resolution generally depends on the number of MEG sensors and the signal source estimation method. In this study, the signal source estimation results fell within a range from 2.0 to 3.5 mm for both current dipoles of between 50 and 100 nAm (Figure 8). This indicates that accurate signal source estimation was achieved even without a shielded room.

The estimation errors for the frontal and occipital regions were larger compared to the current source estimation errors when the 50 current sources were categorized into five brain regions (Table 2). The study set the current dipole frequencies to 3 Hz (delta), 6 Hz (theta), 11 Hz (alpha), 20 Hz (beta), and 40 Hz (gamma). EEG and MEG typically measure brain activity as either transient evoked responses or steady-state spontaneous activity. Although the phantom signal primarily measured evoked responses, its accurate sinusoidal waveform indicates its potential applicability to spontaneous activity. Representative sensory field responses included the P55m (latency 50 ms, 20 Hz) somatosensory field response, the N1m (latency 100 ms, 11 Hz) auditory field evoked response, the 130 ms (11 Hz) visual field evoked response [16], and the auditory steady-state response (40 Hz) [12]. Spontaneous activity responses included frontal theta [17] and occipital alpha [16]. According to Table 2, the errors for evoked responses—such as the parietal P55m, bilateral N1m, occipital responses, and bilateral auditory steady-state responses (40 Hz)—were all within 3.0 mm. For spontaneous activity, the estimation error for 6 Hz in the frontal region was 3.98 mm, which was larger than for occipital alpha and other regions. Importantly, the 40 Hz temporal results remained unaffected by the 40 Hz noise peak observed in the environmental noise spectral density during the zero-boil-off operation. 

Uehara et al. found no difference in signal source estimation error when increasing the number of sensors to 64 or more using the same dry phantom [18]. However, their study did not involve physically removing sensors, but rather selecting a specific number of sensors for estimation. This suggests that estimation accuracy may depend on sensor placement. As shown in Figure 3, sensors in the MEG system are not evenly distributed across regions. The inferior estimation accuracy in the frontal and occipital regions can be attributed to fewer sensors in these areas (frontal: 7 sensors/17 spaces; occipital: 11 sensors/23 spaces; parietal: 20 sensors/28 spaces). In contrast, Ahonen et al. demonstrated that a planar array of 122 sensors provides spatial resolution more than twice as good as that of 61 axial sensors [19]. The MEG system in this study could be expanded to 128 sensors. Considering the spatial resolution of MEG and previous studies, factors such as the number of sensors and their placement could affect the accuracy of signal source estimation. Optimizing these factors could significantly enhance estimation accuracy.

The developed MEG system incorporates several advanced features that distinguish it from conventional systems. Its key component is the HTSMS that greatly enhances environmental noise reduction. This innovation eliminates the need for a magnetic shield room and reduces the space and installation constraints associated with conventional MEG setups. Unlike large structurally demanding magnetic shield rooms that typically require extensive foundation work, the superconducting magnetic shield in our system provides a lighter and more flexible solution. The compact design and modular configuration of the system is designed to facilitate installation, even at locations other than the ground floor. The overall footprint of the system is smaller, and the modular design facilitates installation in a variety of environments, including off-ground locations. Another major advance is the zero-boil-off system, which significantly reduces liquid helium consumption by recondensing the evaporated gas, representing a major advance. This feature reduces operating costs and maintenance requirements, and is clearly superior to conventional systems that require frequent helium refills. Despite this advantage, however, zero-boil-off systems generate noise from the cooling system, especially in the 35–50 Hz range, as shown in the spectral density measurement (Figure 4). This noise can be mitigated with appropriate filtering techniques, but there is a trade-off when compared to a fully passive cooling system. 

OPM-MEG systems are more portable and do not require cryogenic cooling. Our systems have the advantage of high stability and low sensitivity to external magnetic interference due to superconducting shielding. However, it has drawbacks such as sensitivity problems in noisy environments and susceptibility to temperature variations. The MEG system in this study, although less portable, is more robust in a typical laboratory environment and can improve the accuracy of signal estimations without requiring a zero-field environment. This makes them easier to deploy and suggests that they will be suitable for a variety of clinical and research applications.

## 5. Conclusions

This study provides an overview of a MEG system featuring a superconducting magnetic shield and a zero-boil-off system. Performance evaluation using a dry phantom showed signal source estimation accuracy from 2.5 to 3.5 mm, comparable to typical MEG systems. This indicates that the system can be installed in a standard room without a magnetically shielded room while still delivering satisfactory performance. Future work will focus on increasing the number of sensors and mitigating magnetic noise, especially above 35 Hz, introduced by the zero-boil-off system.

## Figures and Tables

**Table 1:** Comparison of the specification of developed HTSMS-MEG and general SQUID type MEG. HTSMS: high-temperature superconducting magnetic shield. SNS: superconductor/normal-metal/superconductor. SIS: superconductor/insulator/superconductor.

	HTSMS-MEG	General SQUID Type MEG
Detection coil	SNS Type SQUID Sensor Axial first-order differential type gradiometer	SIS Type SQUID Sensor Axial first-order differential or planar type gradiometer
Number of sensors	64 (expanded to 128)	More than 300
Size (Includes magnetically shielded rooms)	Length 1 m × Width 2 m × Height 2 m	More than 3 m scale (cube-type 27 cm^3^)
Weight	2000 kg	More than 10,000 kg
Zero-boil-off system in operation	Operable during measurement	Operation stopped during measurement

**Table 2:** Mean source estimation error (mm) for 50 current sources divided into five brain regions.

Region	Dipole	3 Hz	6 Hz	11 Hz	20 Hz	40 Hz
Frontal	50 nAm	4.53	3.98	2.94	2.82	4.27
	100 nAm	2.77	4.02	2.77	3.15	3.29
Left-temporal	50 nAm	3.10	2.45	2.5 7	2.41	2.91
	100 nAm	2.48	2.38	2.42	2.62	2.91
Right-temporal	50 nAm	2.07	2.64	2.05	2.05	2.8 4
	100 nAm	1.74	2.72	1.98	2.20	2.70
Parietal	50 nAm	3.27	2.33	1.29	1.69	2.76
	100 nAm	1.88	2.58	1.46	1.43	2.00
Occipital	50 nAm	2.34	3.24	**2.88**	3.32	4.23
	100 nAm	2.78	3.89	3.11	3.02	3.47

50 current sources (Frontal: 10 sources, Left-temporal: 10 sources, Right-temporal: 10 sources, Parietal: 10 sources, Occipital: 10 sources). Red indicates the result of a signal source, assuming an evoked response. Bolded numbers show the results of the signal source, assuming a spontaneous response.

## Data Availability

The data that support the findings of this study are available from the corresponding author, K.T., upon reasonable request.
